# Plasma levels of soluble PD-1, TIM-3, LAG-3 and galectin-3 and the degree of liver fibrosis in CHC and the impact of successful antiviral treatment on their levels

**DOI:** 10.1038/s41598-025-99096-4

**Published:** 2025-05-02

**Authors:** Sylwia Osuch, Aleksandra Kumorek, Paweł Kozłowski, Hanna Berak, Anna Maria Kochanowicz, Kamila Cortés-Fendorf

**Affiliations:** 1 Department of Immunopathology of Infectious and Parasitic Diseases, Medical University of Warsaw, 3c Pawińskiego Street, Warsaw, 02-106 Poland; 2 Central Laboratory, University Clinical Centre of Medical University of Warsaw, Warsaw, Poland; 3 Outpatient Clinic, Warsaw Hospital for Infectious Diseases, Warsaw, Poland

**Keywords:** SPD-1, STIM-3, SLAG-3, Gal-3, Chronic hepatitis C, Liver fibrosis, Antiviral treatment, Biomarkers, Medical research, Molecular medicine, Pathogenesis

## Abstract

Chronic hepatitis C (CHC), caused by the hepatitis C virus, commonly leads to liver fibrosis. CHC is also related to T-cell exhaustion, phenotypically manifesting as overexpression of inhibitory receptors (iRs), e.g., programmed death receptor-1 (PD-1), T cell immunoglobulin and mucin domain-containing protein 3 (TIM-3) and lymphocyte activation gene 3 (LAG-3), which have corresponding plasma-soluble analogs. Galectin-3 (Gal-3) is a pro-fibrotic and pro-inflammatory molecule, but its role in CHC is controversial. The study aimed to assess the relationship between plasma levels of soluble PD-1 (sPD-1), sTIM-3, sLAG-3 and Gal-3 and the degree of fibrosis in CHC and successful CHC treatment effect on these markers. The study comprised 98 CHC patients, qualified for treatment with direct-acting antivirals. Plasma samples were collected prior to and six months post-treatment. iRs were determined by ELISA. sPD-1 levels were significantly higher in more advanced fibrosis (F2 + F3 vs. F0/1). Regardless of the degree of fibrosis, sPD-1 and sLAG-3 levels significantly decreased after therapy. sTIM-3 levels also decreased, however, mostly in patients with no or mild (i.e., F0/1) fibrosis. Furthermore, Gal-3 increased in patients with more advanced fibrosis (F2 + F3). sPD-1 is associated with liver disease stage in CHC and effective treatment is related to the iRs levels reduction.

## Introduction

Chronic hepatitis C (CHC), caused by the hepatitis C virus (HCV), may lead to liver fibrosis (LF), cirrhosis, and hepatocellular carcinoma (HCC)^1^. Patients who have overcome the infection and cleared the virus are not protected against re-infection^2^. Moreover, no vaccine to prevent HCV infection is yet available, despite continuing efforts to its development^3^.

LF is an excessive accumulation of extracellular matrix proteins, including collagen, reflecting tissue scarring and disease progression in CHC^4^. The degree of LF is critical for the prognosis of infected patients^5,6^. The immune response to HCV infection plays a key role in the development of fibrosis, mainly by stimulation of factors promoting cell proliferation, as well as secretion of profibrogenic cytokines, such as TGF-β1 and RANTES, which intensify the production of extracellular matrix^7,8^. Lymphocytes were also shown to be involved^5,9–11^. Polarized Th2 cells stimulate expression of profibrogenic factors in myofibroblasts (procollagen I and II, MMP-2, MMP-9, TIMPs) and the synthesis of immunoregulatory mediators in macrophages (IL-10, TGF-β)^12,13^.

Human galectin-3 (Gal-3), a β-galactoside-binding lectin^14,15^ is a pro-fibrotic molecule produced by different cells such as leukocytes, epithelial and endothelial cells^16^. It modulates fibroblasts and macrophages activity in chronically inflamed organs, affecting fibroproliferative pathways promoting fibrosis^17,18^. Mice deficient in Gal-3 were protected from carbon tetrachloride- induced LF^14^ and non-alcoholic steatohepatitis (NASH)^19,20^. The evidence supporting a causal function of Gal-3 in tissue fibrosis led to the development of its blocking agents as anti-fibrotic drugs^21^.

Although non-invasive techniques assessing LF such as transient elastography (TE) have become routine, validated procedures in the setting of CHC patients care, cheap, sensitive, specific, reliable serum surrogate biomarkers could be invaluable in remote and/or resource-limited healthcare settings, with limited access to this procedure^22^. Current non-invasive biomarkers in routine use represent mainly indirect markers associated with liver damage and/or deterioration of liver function, such as ALT, AST, gamma-glutamyltransferase (GGT), bilirubin, albumin, platelet count, prothrombin time, globulin, glucose, insulin, apolipoprotein, cholesterol and haptoglobin, which are not sufficiently sensitive and specific^23,24^. Their combinations in composite algorithms such as FIB-4, APRI scores were shown to have high negative predictive value (NPV) and are hence useful for excluding a diagnosis of advanced LF, however, they perform poorly at identifying patients with the disease^25,26^.

Immune exhaustion is an immune system cell dysfunction characteristic of chronic infections including HCV infection and cancer, in which cells are exposed to constant antigenic stimulation and inflammatory signals^27,28^. Antigen level and the time of exposure are the main promoting factors^29^. It mainly affects T-cells, although was also observed in NK cells or B-cells^30,31^. In particular, CD8^+^T-cells undergo a progressive loss of effector functions, ranging from impaired proliferation and the ability to secrete effector cytokines, to the loss of cytotoxicity as well as altered transcriptional program, which is accompanied by an upregulated expression of “inhibitory” receptors (iRs), mainly represented by programmed death receptor-1 (PD-1), T-cell immunoglobulin and mucin domain-containing protein 3 (TIM-3), lymphocyte activation gene 3 (LAG-3)^27,28,32^. Progression of exhaustion is characterized by excessive, constitutive, simultaneous expression of multiple iRs, which interact with their respective ligands on the surface of antigen-presenting cells (APCs) having negative effects on T-cell proliferation, cytokine secretion, metabolism and survival^33–42^.

LF impairs the homeostatic role of the liver in the systemic immune response and has been associated with a skewed immune profile^43,44^. In CHC, surface iRs expression has been correlated with disease progression and poor prognosis. For example, LF severity in CHC was associated with hyperfunctional CD8^+^T-cell responses, characterized by increased iR expression and systemic inflammation^45^. Furthermore, in spleen and peripheral blood of CHC patients with cirrhosis and portal hypertension CD4^+^ and CD8^+^T-cells highly expressed PD-1 and Tim-3^46^.

We also have previously shown that patients with more advanced LF (i.e., F2-F3) had higher PD-1 and lower TIM-3 expression on CD4^+^T-cells and treatment had little or no effect on the exhaustion markers in these patients^47^. These findings suggest a potential involvement of immune exhaustion in the immunopathology of liver disease.

In addition to the membrane-bound molecules, soluble PD-1, TIM-3, as well as LAG-3 are detectable in plasma, mainly as a result of enzymatic cleavage from the cell surface by metalloproteinases, cells breakdown, or alternative splicing^48–51^. sPD-1 was shown to compete with membrane PD-1, reducing inhibitory signaling to T cells, partially restoring immune function and exacerbating inflammatory responses^52^. sTIM-3 can disrupt interaction between the surface TIM-3 and its ligand, partially alleviating T-cell exhaustion, restoring their ability to proliferate and produce pro-inflammatory cytokines^53^. Thus, their impact may be dual: protective, by enhancing immune cell function, and detrimental, by exacerbating inflammation and thus liver injury. However, it has not been investigated whether the levels of soluble iRs correlate with the severity of liver disease in the course of CHC and whether successful antiviral treatment based on direct-acting antivirals (DAAs) affects their plasma levels. This is especially important, since these molecules, with their potentially immunoregulatory properties, may be involved in the pathogenesis of fibrosis in CHC, but also may be considered potential non-invasive plasma markers of HCV-related disease progression.

The aim of the study was to assess the relationship between the levels of sPD-1, sTIM-3 and sLAG-3 as well as Gal-3 in plasma and the degree of LF in patients with CHC, as well as to investigate the impact of effective DAA-based treatment on their levels.

## Materials and methods

Plasma samples from 98 patients with CHC, qualified for antiviral treatment at the Warsaw Hospital for Infectious Diseases were prospectively collected right before treatment initiation and 6 months after treatment completion. The inclusion criteria were chronic HCV infection with genotype 1b, no other clinically relevant viral infections (HIV-1, HBV), and no HCC diagnosis. Twenty-three patients were treated with the combination of Viekirax (AbbVie) (ombitasvir + paritaprevir + ritonavir) and Exviera (AbbVie) (dasabuvir), 68 received Harvoni (Gilead) (ledipasvir + sofosbuvir) and the remaining seven received Zepatier (MSD) (elbasvir + grazoprevir), according to the standard recommendations. All treatments lasted 12 weeks, except for 32 Harvoni-treated patients and seven treated with Vekirax and Exviera, as they lasted 8 weeks. HCV genotype was determined using the Inno-LIPA HCV II test (Innogenetics). Baseline viral titer was determined using the Abbott HCV RealTime assay (sensitivity 12 IU/ml). The degree of LF was assessed by TE method and classified according to the Metavir scale in which F0/F1 represents no or minimal fibrosis (*n* = 56), F2 moderate fibrosis (*n* = 27), F3 - severe fibrosis (*n* = 15), F4 - cirrhosis (*n*= 0)^22^. Moreover, clinical data were collected, such as: baseline age, sex, viral load and ALT. The characteristics of the study group are presented in the Table 1.

All methods were performed in accordance with the guidelines of the 2013 Declaration of Helsinki. Written informed consent was obtained from all patients prior to the study initiation. The experimental protocol was approved by the Bioethics Committee of the Medical University of Warsaw (consent numbers KB/77/A/2015 and AKBE/265/2023). Assessment of sPD-1, sTIM-3 and LAG-3 in plasma was conducted using “Human PD-1 ELISA Kit”, “Human TIM-3 ELISA Kit” and “LAG-3 Human ELISA Kit”, respectively (all from Thermo Fisher Scientific) according to the manufacturer’s protocol. “Human Galectin-3 ELISA Kit” (Proteintech) was used to assess Gal-3 levels. Calculations of sPD-1 sTIM-3, LAG-3 and Gal-3 plasma levels were performed using the www.MyAssays.com program, employing a 5-line regression model to plot a standard curve. The results were expressed in pg/ml as medians (range). The Kruskal-Wallis and Mann-Whitney tests were used to make comparisons of plasma concentrations of the analyzed markers before treatment between groups representing different degrees of LF. The Wilcoxon matched-pairs signed-ranks test was used to assess the treatment effect on the analyzed markers within each fibrosis group. Spearman nonparametric correlation was used to test whether analyzed markers plasma levels correlate with viral load, ALT levels, age, as well as with each other.

The fibrosis grade prediction performance of each marker was assessed using the receiver operating characteristics curves (ROC). The area under the curve (AUC) with 95% confidence interval (CI) was calculated; good performance was defined as AUC > 0.8^54^. The prediction sensitivity and specificity of each biomarker were also calculated.

The statistical comparisons, correlations and ROC curves were performed using the InStat program (GraphPad Software, USA). All P-values were two-tailed and considered significant when < 0.05.

**Table 1:** Clinical and epidemiological characteristics of the study participants.

Liver Fibrosis (Metavir scale*)	F0/1 (*n* = 56)	F2 (*n* = 27)	F3 (*n* = 15)	F2 + F3 (*n* = 42)	All (F0/1 + F2 + F3) (*n* = 98)
**Sex** [F/M]	41/15	12/15	9/6	21/21	62/36
**Age**					
Median (range) mean ± SD	53.5 (25.0–83.0) 53.1 ± 16.0	61.0 (28.0–81.0) 57.3 ± 14.1	60.0 (34.0–88.0) 58.8 ± 14.1	60.0 (28.0–88.0) 57.7 ± 13.9	57.5 (25.0–88.0) 55.1 ± 15.3
**ALT [U l** ^**−1**^ **]**					
Median (range) mean ± SD Normal values: *7–56* U/l	56.0 (19.0–255.0) 62.7 ± 33.7	65.0 (33.0–389.0) 102.7 ± 87.9	69.0 (29.0–199.0) 95.3 ± 102.7	66.5 (29.0–389.0) 100.1 ± 76.6	61.0 (19.0–389.0) 78.7 ± 58.9
**Baseline viral load ** **[IU ml** ^**−1**^ **]**					
Median (range) mean ± SD	5.4×10^5^ (6.2×10^3^−4.9ˣ10^6^) 9.6×10^5^ ± 1,1×10^6^	9.3×10^5^ (5.2×10^4^ − 1.1×10^7^) 1.8×10^6^ ± 2.4×10^6^	2.1×10^6^ (4.8×10^5^ − 6.7×10^6^) 2.7×10^6^ ± 1.9×10^6^	1.2×10^6^ (5.2×10^4^ − 1.1×10^7^) 2.1×10^6^ ± 2.3×10^6^	8.4×10^5^ (6.2×10^3^ − 1.1×10^7^) 1.4×10^6^ ± 1.8×10^6^
**DAA treatment scheme**:					
ombitasvir + paritaprevir + ritonavir + dasabuvir ledipasvir + sofosbuvir elbasvir + grazoprevir	6 44 6	9 18 0	8 6 1	17 24 1	23 68 7

* F0/F1 represents no or minimal fibrosis, F2 moderate fibrosis, F3 severe fibrosis^22^.

## Results

### sPD-1 levels are increased in patients with more advanced LF

**Fig. 1:**
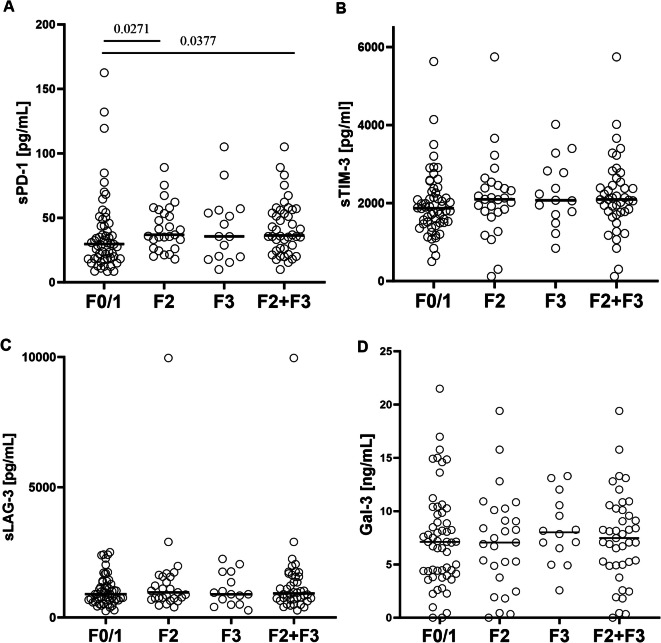
sPD-1, sTIM-3, sLAG-3 and Gal-3 plasma levels in CHC patients with varying degrees of LF according to the Metavir score. F0/F1 represents no or minimal fibrosis, F2 moderate fibrosis, F3 - severe fibrosis^22^. The horizontal lines denote the median value, while the statistically significant P values ​​are indicated above the lines representing pairwise comparisons.

sPD-1 plasma levels were 29.7 (8.4–162.6), 36.8 (18.0–89.1) and 35.6 (10.0–105.1) pg/ml [median (range)] in patients with F0/1, F2 and F3 score, respectively and 36.3 (10.0–105.1) pg/ml in the combined F2 and F3 group (Fig. 1A). Importantly, sPD-1 levels were significantly higher in the F2 and F2 + F3 subgroups than in the F0/1 group (*P* = 0.0271 and *P* = 0.0377, respectively).

sTIM-3 plasma levels were 1,868.5 (500.9-5,627.5), 2,094.9 (120.8-5,746.8) and 2,071.4 (839.4-4,014.9) pg/ml in patients with F0/1, F2 and F3 score, respectively, and 2,090.6 (120.8-5,746.8) pg/ml in the combined F2 and F3 group (Fig. 1B). No statistically significant differences were observed between these groups (Fig. 1B).

sLAG-3 plasma levels were 896.2 (244.3-2,503.0), 964.1 (392.2-9,962.0) and 892.4 (276.9-2,248.0) pg/ml in F0/1, F2 and F3 patients, respectively, and 922.4 (276.9-9,962.0) pg/ml in the combined F2 and F3 groups (Fig. 1C). No statistically significant differences were observed between these groups (Fig. 1C).

Gal-3 plasma levels were 7.1 (0.0–21.5), 7.1 (0.0–19.4) and 8.0 (2.6–13.3) pg/ml in F0/1, F2 and F3 patients, respectively, and 7.5 (0.0–19.4) ng/ml in the combined F2 and F3 groups (Fig. 1D). No statistically significant differences were observed between these groups (Fig. 1D).

## sPD-1 positively correlates with HCV viral load, ALT and sLAG-3 levels in CHC

**Fig. 2:**
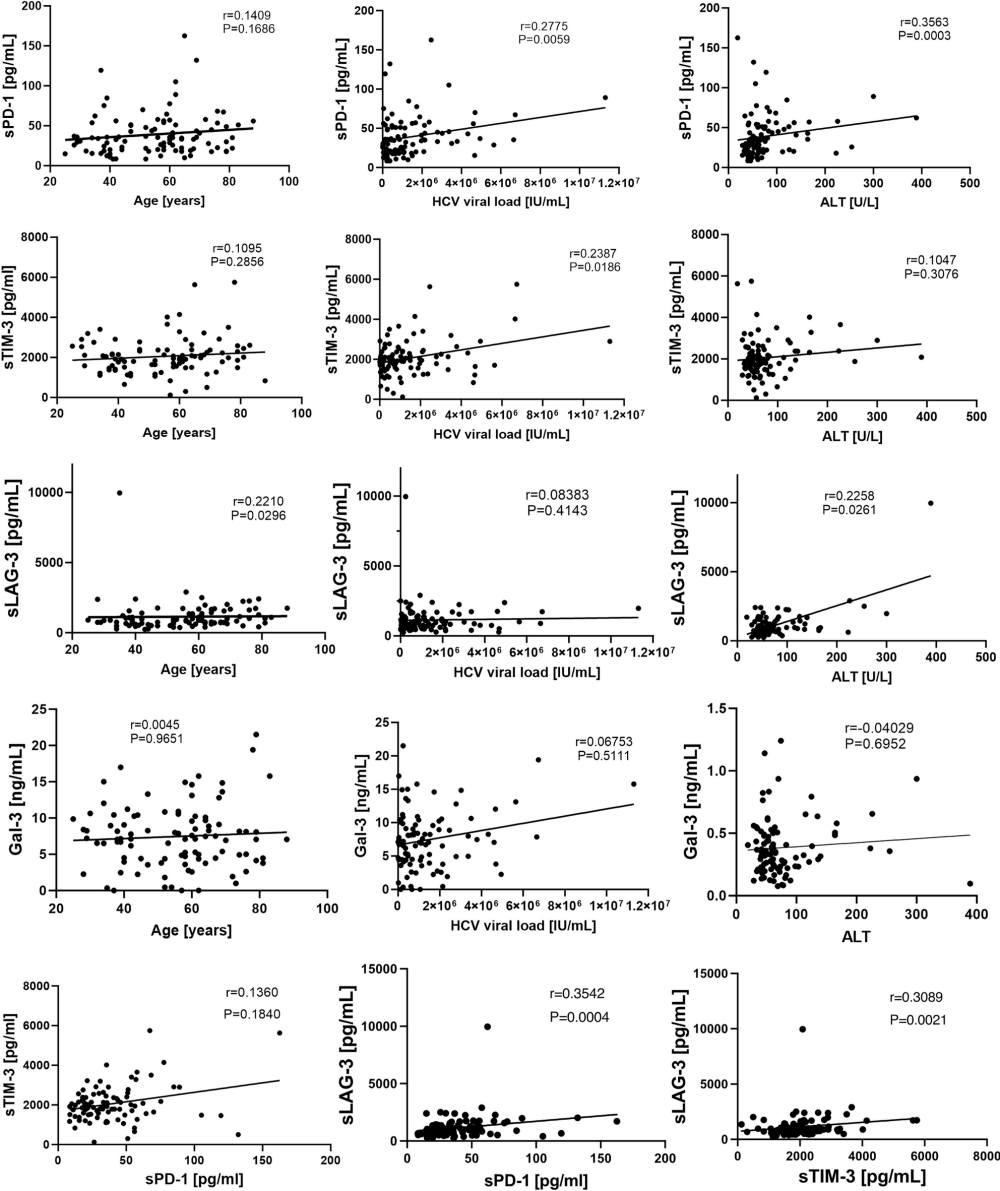
Correlation analysis of pretreatment sPD-1, sTIM-3, sLAG-3 and Gal-3 and the clinical parameters of the study cohort.

Pretreatment sPD-1 levels in plasma were found to positively correlate with baseline viral load (*r* = 0.2775, *P* = 0.0059), baseline ALT levels (*r* = 0.3563, *P* = 0.0003) as well as with LAG-3 (*r* = 0.3542, *P* = 0.0004), Fig. 2. Plasma sTIM-3 positively correlated with baseline HCV viral load *r* = 0.2387, *P* = 0.0186, and sLAG-3 (*r* = 0.3089, *P* = 0.0021). sLAG-3 positively correlated with baseline ALT levels *r* = 0.2258, *P* = 0.0261, as well as with baseline age (*r* = 0.2210, *P* = 0.0296). We found no correlation between galectin-3 levels and neither of the analyzed parameters (Fig. 2).

## Successful treatment of CHC is associated with reduction of soluble iR levels in plasma

**Fig. 3:**
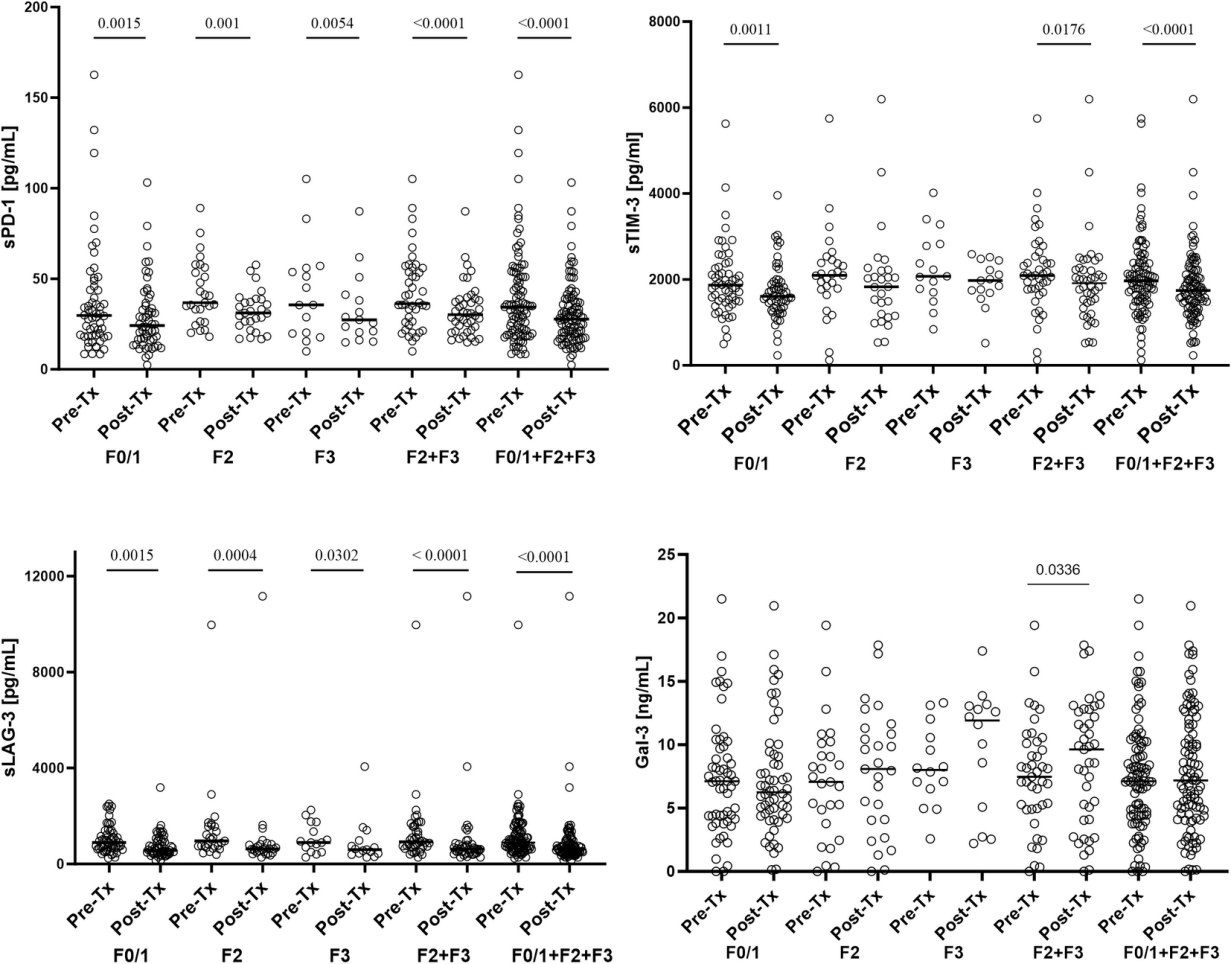
Effect of successful treatment on plasma sPD-1, sTIM-3, sLAG-3 and Gal-3 levels in patients with various degrees of LF. F0/F1 represents no or minimal fibrosis, F2 moderate fibrosis, F3 severe fibrosis according to the Metavir score^22^. The horizontal line denotes median, while statistically significant P values ​​are indicated above the lines representing pairwise comparisons.

Before treatment, median sPD-1 level in all combined groups of LF (F0/1 + F2 + F3) was 34.3 (8.4–162.6) pg/ml and decreased to 27.7 (2.4–103.2) pg/ml after treatment (*P* < 0.0001); (Fig. 3). A statistically significant decrease in sPD-1 levels was also observed in all particular fibrosis groups (i.e., from 29.7 (8.4–162.6) pg/ml to 24.2 (2.4–103.2) pg/ml in F0/1, *P* = 0.0015; from 36.8 (18.0–89.1) pg/ml to 31.1 (16.7–57.7) pg/ml in F2, *P* = 0.001); from 35.6 (10.0–105.1) pg/ml to 27.3 (14.9–61.9) pg/ml in F3; *P* = 0.0054; and from 36.3 (10.0–105.1) pg/ml to 30.2 (14.9–61.9) pg/ml in F2 + F3 group; *P* < 0.0001); (Fig. 3).

Before treatment, sTIM-3 level in F0/1 + F2 + F3 combined groups was 1,966.8 (120.8-5,746.8) pg/ml, and significantly decreased after treatment to 1,742.1 (235.7-6,195.1) pg/ml, *P* < 0.0001); (Fig. 3). Similarly, in the F0/1 and F2 + F3 groups, a statistically significant decrease in sTIM-3 concentration was observed (from 1,868.5 (500.9-5,627.5) pg/ml to 1608.5 (235.7- 3,959.0) pg/ml, *P* = 0.0011 and from 2,090.6 (120.8-5,746.8) pg/ml to 1,912.3 (518.5-6,195.1) pg/ml, *P* = 0.0176, respectively (Fig. 3). In individual F2 and F3 groups, a decrease in sTIM-3 concentration was also observed (from 2,094.9 (120.8-5,746.8) pg/ml to 1827.5 (533.3-6,195.1) pg/ml and from 2,071.4 (839.4-4,014.9) pg/ml to 1,974.5 (518.5-2,521.0) pg/ml, respectively, however, the observed differences were not statistically significant (Fig. 3).

Before treatment, sLAG-3 levels in F0/1 + F2 + F3 combined groups was 896.2 (244.3-9,962.0) pg/ml, and significantly decreased after treatment to 609.0 (163.8–11,156.0) pg/ml, *P* < 0.0001). In all particular groups, a statistically significant decrease in sLAG-3 levels was observed (from 896.2 (244.3- 2,503.0) to 570.1 (163.8-3,189.0) pg/ml in F0/1, *P* < 0.0001); from 964.1 (392.2- 9,962.0) to 629.4 (280.2–11,156.0) pg/ml in F2, *P* = 0.0004; from 892.4 (276.9-2,248.0) to 598.0 (279.4-4,062.0) pg/ml in F3, *P* = 0.0302; and from 922.4 (276.9–9,962.0) to 621.9 (279.4–11,156.0) pg/ml in F2 + F3 group; *P* < 0.0001); (Fig. 3).

Before treatment, median Gal-3 levels in F0/1 + F2 + F3 combined groups was 7.1 (0.0–21.5) ng/ml and did not significantly change after treatment (7.2 (0.0–21.0) ng/ml). In F0/1 group, Gal-3 levels tended to decrease (from 7.1 (0.0–21.5) to 6.2 (0.1–30.0) ng/ml, while in individual F2 and F3 groups these tended to increase (from 7.1 (0.0–19.4) to 8.1 (0.0–17.8) and from 8.0 (2.6–13.3) to 11.9 (2.2–17.4) ng/ml, respectively). However, the observed differences did not reach statistical significance. A statistically significant increase in galectin-3 levels was observed in combined F2 + F3 groups (from 7.5 (0.0–19.4) to 9.6 (0.0–17.8) ng/ml, *P* = 0.0336, Fig. 3).

## **Performance of plasma sPD-1**,** sTIM-3 sLAG-3 and Gal-3 as potential LF markers in CHC**

**Fig. 4:**
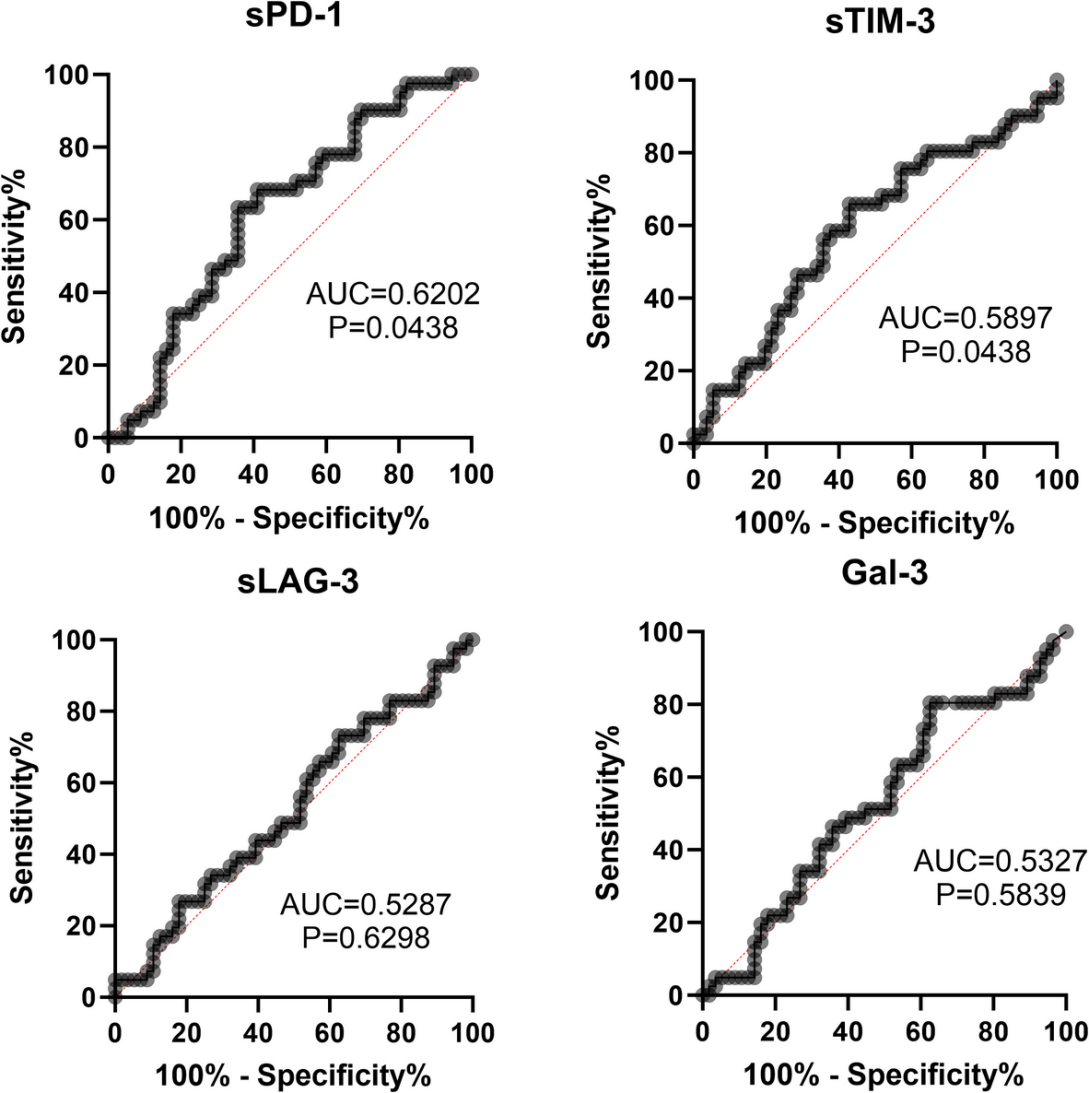
ROC curves for predicting advanced fibrosis (i.e., F2/F3 vs. F0/1 according to the Metavir score) of sPD-1, sTIM-3, sLAG-3 and Gal-3.

We tested the performance of the analyzed molecules to predict fibrosis grade in CHC patients using the ROC curves (Fig. 4). The AUC of PD-1 for detection of more advanced fibrosis (i.e., F2/F3 vs. F0/1) was 0.6202 (95% CI: 0.5084 to 0.7320, *P* = 0.0438) and the cut-off value of > 35.0 pg/ml could predict higher fibrosis with sensitivity of 63.4% (95% CI: 48.1–76.4%) and specificity of 64.3% (95% CI: 51.2–75.5%).

For sTIM-3, the analogic AUC was 0.5897 (95% CI: 0.4733 to 0.7060, *P* = 0.1325) and the cut-off value of > 1918.0 pg/ml could predict higher fibrosis with sensitivity of 65.8% (95% CI: 50.5–78.4%) and specificity of 57.1% (95% CI: 44.1–69.2%).

For sLAG-3, the AUC was 0.5287 (95% CI: 0.4117 to 0.6457, *P* = 0.6298) and the cut-off value of > 809.3 pg/ml could predict higher fibrosis with sensitivity of 61.0% (95% CI: 45.7–74.3%) and specificity of 46.4% (95% CI: 34.0–59.3%).

For Gal-3, the AUC was 0.5327, (95% CI: 0.4158 to 0.6496, *P* = 0.5839) and the cut-off value of > 7.3 pg/ml could predict higher fibrosis with sensitivity of 51.2% (95% CI: 36.5–65.7%) and specificity of 55.4% (95% CI: 42.4–67.6%).

## Discussion

The aim of the presented study was to assess the relationship between the sPD-1, sTIM-3 sLAG-3 and Gal-3 in plasma and the degree of LF in patients with CHC, and to investigate the effect of successful antiviral treatment on these markers. So far, it has not been investigated whether the level of soluble forms of these receptors is related to the severity of liver disease during CHC and whether treatment-driven viral eradication affects their levels. A characteristic feature of sPD-1, sTIM-3 sLAG-3 and Gal-3 is an easy detection in readily available material, such as plasma, without the need of fresh or cryoprotected cells, and more complicated and costly determination of the membrane forms of both receptors using the flow cytometry method. Therefore, there is a clear potential of soluble iRs as markers of liver disease progression in CHC.

The results of the presented study showed a significantly higher concentration of sPD-1 in plasma of patients with more advanced LF (i.e., F2 and F2 + F3) than in patients with no fibrosis or with a low degree of fibrosis (F0/1). We have previously shown that patients with advanced LF had higher percentages of peripheral PD-1^+^CD4^+^T-cells than patients with less pronounced fibrosis^47^. Now we extrapolate this finding to the soluble PD-1 in plasma. We found no similar studies reported for HCV infection, but patients with chronic hepatitis B (CHB) showed higher levels of sPD-1 than in the control group and in those who eliminated the virus spontaneously. Similarly, an increase in sPD-1 level was associated with the severity of inflammation and the degree of LF^55–57^. Our study may imply that sPD-1 may be involved in the immunopathogenesis of LF, but the mechanism behind this observation remains to be determined. It was previously shown that sPD-1 may neutralize monoclonal antibodies against membrane iRs used in cancer immunotherapy^58^. Moreover, it may have itself an immunoregulatory effect^49^. Potentially, soluble, circulating receptors may exhibit cytokine-like properties, modulating function of other cells of the immune system^48^. Soluble iRs were shown to bind ligands on APCs surface, thereby blocking the interaction of membrane-bound “inhibitory” receptors with these ligands^49^. For example, sPD-1 blocks the PD-1/PD-L1 signaling pathway by a negative feedback mechanism, thus an increase in sPD-1 concentration reduces the inhibition of T-cell activation, which could theoretically improve their functionality, including the cytotoxic effect to HCV-infected hepatocytes, and potentiated fibrosis^59–62^. Our finding of positive correlation between sPD-1 levels and ALT as well as HCV viral load could also support this hypothesis.

We also tested the potential of sPD-1 as an easily quantifiable marker of liver disease progression in the course of CHC, but the performance to discriminate mild (i.e., F0/1) from more advanced fibrosis (i.e., F2/F3) was relatively poor (AUC < 0.8). Possibly, it might have been better if the study included also patients who display the F4 grade.

Contrary to sPD-1, the median level of sTIM-3, LAG-3 and Gal-3 in plasma were shown to be similar in all groups of patients (F0/1, F2, F3, F2 + F3), which indicates their limited involvement in the immunopathogenesis of LF as well as potential as markers of LF stage of in the course of CHC. Although there is very limited data for comparison in CHC, similar to the results obtained in the presented study, no correlation was found between the level of sTIM-3 and the histopathological degree of LF in patients with autoimmune hepatitis^63^. Similarly, although we could not find analogic data in CHC-related liver disease, serum levels of LAG-3 were significantly elevated in HCC patients when compared to healthy controls, but the enrolled patients were prevalently HBsAg-positive^64^. LAG-3 expression levels were also found to be elevated on CD8^+^intrahepatic and peripheral T-cells in CHC when compared to HCV-negative patients, which was related to deteriorated effector functions of these cells^65^. Notably, F0-F2 and F3-F4 patients showed no differences in frequencies of LAG-3^+^ CD8^+^ and CD4^+^T-cells^66^.

In case of plasma Gal-3, disparate results have been previously reported. CHC patients were shown to have similar^67^ or even lower serum Gal-3 than healthy controls^68^. Similarly, Gal-3 did not correlate with the degree of LF, viral load, viral genotype, CRP, leukocyte count, or the model for end stage liver disease (MELD) score in CHC^69^. Our study also did not find correlation neither between Gal-3 and HCV viral load, nor ALT and patients’ age.

In other studies, cirrhotic CHC patients were found to have higher serum Gal-3 levels than the corresponding CHB patients^70^. Similarly, there was no relationship between the Gal-3 and the fibrosis score in CHB^71^. Thus, these viral infections seem to affect serum Gal-3 levels independent of the fibrosis stage and seem not to be closely linked with the extent of liver dysfunction in CHC.

The second research goal was to assess the effect of successful DAA treatment of CHC on plasma level of the analyzed molecules. Contrary to IFN-α therapy, which may directly affect the concentration of the tested markers because of immunomodulatory effect, DAA treatment is not directly immunomodulatory^72^, thus it offers a unique and convenient model to test this effect.

Effective treatment, through viral eradication and the resulting arrestment of chronic antigenic stimulation, may contribute to reduction of plasma iRs. On the other hand, patients with CHC-related LF may display immune dysregulation, which persists after viral clearance. It was previously shown that bulk CD8^+^T-cells of CHC patients with advanced liver disease were dysregulated and showed sustained activated status compared to those with minimal LF, and this status persisted post-DAA treatment^45^. Importantly, these differences were present not only in CHC patients relative to healthy individuals, but also in patients with advanced fibrosis compared to those with minimal or no fibrosis. The presented study has showed a statistically significant decrease in sPD-1 concentration after successful treatment in the combined group of all studied patients (F0/1 + F2 + F3). These results point to the elimination of HCV as the cause of the decrease in sPD-1 levels and confirm that these may be reduced. Moreover, stratification into groups of varying degrees of LF also revealed a statistically significant decrease in sPD-1, suggesting that this effect may occur at various stages of HCV-related liver disease, including the advanced stage (F3). Successful treatment was previously shown to contribute to the improvement of the exhaustion phenotype of T-cells^38^. In particular, we previously showed that the percentages of CD4^+^ and CD8^+^T-cells with TIM-3 expression as well as PD-1 and TIM-3 co-expression decreased to levels similar to those in healthy subjects, which was accompanied by a reduction of immunosuppressive IL-10 in plasma^47^. In addition, DAA-mediated HCV antigen removal restored the function of HCV specific CD8^+^T-cells, including decreased levels of PD-1 expression^73^. Similar findings were observed in studies conducted in patients with CHB, in which serum sPD-1 and sPD-L1 decreased following antiviral therapy associated with suppression of HBV replication^74,75^. sPD-1 levels have also reduced following antiretroviral therapy for primary HIV-1 infection^49^.

Analogous to sPD-1, we also observed a reduction of sLAG-3, both in case of entire cohort group, as well as in each particular LF subgroups, indicating a beneficial effect of treatment at various stages of HCV-related LF. Although we could not find a similar study in CHC, a cross-sectional study employing HIV-1 model revealed that sLAG-3 levels were found to be similar in uninfected individuals and people living with HIV (PLH) on-ART, but were significantly elevated in PLH off-ART^76^.

Plasma sTIM-3 also statistically significantly decreased after successful treatment in the group of all studied patients (combined F0/1 + F2 + F3). There are no similar studies in the HCV model of infection, but cross-sectional HIV-1 studies have shown similar findings: in those receiving long-term antiretroviral therapy, which suppressed viral replication, sTIM-3 levels were lower than in those not receiving treatment^48^. Similarly, in an observational study, sTIM-3 levels correlated with viral load levels and decreased after initiation of antiretroviral treatment^48,77^. Contrary to the F0/1 group, in the F2 and F3 groups, a reduction in sTIM-3 was not observed. This may indicate that, in more advanced liver disease, sTIM-3 levels are less prone to reduction following HCV elimination.

Our study did not show significant reduction in Gal-3 levels following HCV eradication. Interestingly, while in F0/1 subgroup it tended to decrease, in F2 + F3 subgroup it significantly increased. This was the putative reason why in the entire cohort we did not find the significant effect, unlike it was showed in another study^69^. The clinical significance of the post-treatment increase of Gal-3 in patients with more advanced fibrosis remains to be determined.

Despite DAA-driven HCV elimination, inflammatory changes in the liver may still progress, including the risk of HCC development^45,78,79^. The causes of this phenomenon are not fully understood, but are believed to be immune-related^45^. For this reason, prognostic markers of HCC development of HCV etiology are urgently needed. The utility of the same analyzed markers has been already shown elsewhere^58,80–86^. Oncologic treatment employing immune checkpoint inhibitors (ICI) via blockade of interactions between the iRs and their ligands was shown to enhance immune function and has become a standard of care in numerous cancer entities^87^. The presented study, in a longer perspective, might be a starting point of assessing the potential of such markers in HCC prognosis and treatment individualization.

Despite best efforts, our study has shortcomings. First of all, while the analyzed molecules represent easily quantifiable laboratory biomarkers, these may not exactly reflect the cellular expression of the iRs. Thus, their use in the description of the exact picture of immune exhaustion might be limited. Second, in order to diminish eventual confounding effect of HCV genotypes heterogeneity, the study included exclusively 1b – accounting to approximately 82% of infections in Poland as well as majority of infections in Central Europe^88,89^. Thus, the observations made herein may not be extendable to other HCV genotypes.

Third, our study did not employ patients with cirrhosis (F4 score) because of limited access to these patients’ samples. Further prospective studies employing this clinical condition are needed to reflect the analyzed aspect from a wider perspective.

## Conclusions

In summary, the obtained results were novel and allowed for a characterization of the analyzed markers in LF of CHC origin. In particular, sPD-1 showed a potential for easily quantifiable marker of liver disease staging of HCV etiology since its concentration was shown to increase in groups with more advanced liver histopathological changes. In addition, the use of the DAA-mediated treatment model allowed to confirm the hypothesis that treatment-driven viral eradication may be associated with the reduction of the analyzed markers levels in plasma, which occurred for sPD-1 and sLAG-3, regardless of the liver disease staging. In a longer perspective, the presented study can be used as a starting point to validate the potential of the analyzed molecules as prognostic markers of HCC development of HCV etiology.

## Data Availability

All data generated or analysed during this study are included in this published article.
